# Evaluating the cross-cultural validity of the Dutch version of the Social Exclusion Index for Health Surveys (SEI-HS): A mixed methods study

**DOI:** 10.1371/journal.pone.0224687

**Published:** 2019-11-05

**Authors:** Addi P. L. van Bergen, Annelies van Loon, Matty A. S. de Wit, Stella J. M. Hoff, Judith R. L. M. Wolf, Albert M. van Hemert

**Affiliations:** 1 Staff Department, Public Health Service Hollands Midden, Leiden, The Netherlands; 2 Department of Psychiatry, Leiden University Medical Centre, Leiden, The Netherlands; 3 Department of Epidemiology, Health Promotion and Care Innovation, Public Health Service of Amsterdam, Amsterdam, The Netherlands; 4 Department of Income and Social Security, The Netherlands Institute of Social Research|SCP, The Hague, The Netherlands; 5 Impuls, the Netherlands Center for Social Care Research, Radboud University Medical Center, Nijmegen, The Netherlands; Chinese Academy of Medical Sciences and Peking Union Medical College, CHINA

## Abstract

**Background:**

The recently developed Social Exclusion Index for Health Surveys (SEI-HS) revealed particularly strong social exclusion in non-Western immigrant groups compared to the native Dutch population. To qualify such results, cross-cultural validation of the SEI-HS in non-Western immigrant groups is called for.

**Methods:**

A sequential explanatory mixed methods design was used, employing quantitative data from the Netherlands Public Health Monitor along with qualitative interviews. Data from 1,803 adults aged 19 years or older of Surinamese, 1,009 of Moroccan and 1,164 of Turkish background and 19,318 native Dutch living in the four largest cities in the Netherlands were used to test the factorial structure of the SEI-HS and differential item functioning across immigrant groups. Additionally, 52 respondents with a high score on the SEI-HS and from different background were interviewed on the item content of the SEI-HS and subjective feelings of exclusion. For each SEI-HS item the semantic, conceptual and contextual connotations were coded and compared between the immigrant groups and native Dutch.

**Results:**

High levels of social exclusion were found in 20.0% of the urban population of Surinamese origin, 20.9% of Moroccan, 28.7% of Turkish and 4.2% of native Dutch origin. The 4-factor structure of the SEI-HS was confirmed in all three immigrant groups. None of the items demonstrated substantial differential item functioning in relation to immigration background. The interviews uncovered some methodological shortcomings, but these did not substantially impact the observed excess of social exclusion in immigrant groups.

**Conclusions:**

The present study provides evidence in support of the validity of the SEI-HS in adults of Surinamese, Moroccan and Turkish background and confirms the major social exclusion of these immigrant groups in the main cities in the Netherlands. Policy measures to enhance social inclusion and reduce exclusion are urgently needed.

## Introduction

Social exclusion (SE) refers to the inability of people to participate fully in the society in which they live [1]. It is characterised by an accumulation of disadvantages on multiple dimensions: 1) social e.g. sense of belonging and social support; 2) economic e.g. material deprivation; 3) political e.g. lack of access to housing and health care; and 4) cultural e.g. acceptance of values, norms and ways of living [2, 3]. SE has a profound impact on people’s lives. Socially excluded persons report feelings of loss and shame, alienation, powerlessness and insecurity [4–6] resulting in loss of aspirations [4], withdrawal [7, 8], reduced self-confidence [6, 8] and high risk behaviour [9, 10]. SE is considered as one of the driving forces of health inequalities [2, 3, 11, 12] and is particularly relevant in the context of immigrant health [13, 14].

In the past decades, the number of immigrants living in Western Europe has increased significantly [15]. In the Netherlands on average 13 per cent of the population is of non-western origin, with higher representation in urban areas [16]. In the four largest cities, Amsterdam, Rotterdam, The Hague and Utrecht, one in three citizens is of non-western origin (34%), with first and second generation immigrants of Surinam, Morocco and Turkey constituting the largest groups (7.3%, 7.7% and 6.1% respectively). Immigration from Morocco and Turkey was initially labour-related dating back to the 1960’s, while the Surinamese immigration is related to the colonial past and had its highest influx in the period before Surinam’s independence in 1975 [17].

The Netherlands is one of the few EU-countries with a strong record of monitoring immigrant health and health related factors [18]. The Dutch Public Health Monitor (PHM), a four-yearly national health survey that routinely includes data on migration background, employs a large stratified sample, includes strategies to enhance response rates in cities with a diverse ethnic makeup and makes use of culturally validated questionnaires [19–23].

In 2012 we developed an index to measure the four dimensions of social exclusion: the Social Exclusion Index for Health Surveys (SEI-HS) [24]. It was developed as an embedded measure using items from the PHM and where the PHM fell short, supplemented with items from the Social Exclusion Index of the Netherlands Institute for Social Research|SCP [20, 24]. The SEI-HS was validated for the adult population of the Netherlands, including 5.2% respondents with a non-western origin [24], but it was not validated specifically for immigrant groups. In cross-cultural research group differences may result from systematic biases in the way people from different cultures respond. Response style behaviour is reported to differ between cultural groups, with non-western immigrants showing higher acquiescence and midpoint responding [25] or preferring extreme categories more than other groups [26]. Additionally, items that contain content or language that is differentially familiar or has a different connotation for various groups may compromise the cross-cultural validity [27].

Particularly high levels of SE were observed in adults of non-western background measured with the SEI-HS in 2012. One in five adults (21.0%) of non-western background was classified as moderate to strong SE, while the prevalence rates in adults of native Dutch and western migration background were 2.7% and 6.5% respectively [28].

Differences in SE might be expected given that risk factors for SE, such as low educational level, low income, low labour market position, linguistic problems and poor health [20], tend to occur more frequently in non-western immigrant groups than in native Dutch and western immigrant groups [29, 30]. The magnitude of the differences was so large, however, that suspicion has been raised on a potential cultural bias of the SEI-HS.

The leading question for the present study was whether the strong SE among adults of Surinamese, Moroccan and Turkish background compared with native Dutch citizens in the four largest cities in the Netherlands, can be explained by shortcomings in the cross-cultural validity of the SEI-HS.

To answer the research question, a mixed methods approach was chosen. In addition to quantitative testing of the cross-cultural validity through confirmatory factor analysis and differential item functioning (DIF) analysis [31], qualitative interviews were conducted with socially excluded respondents of immigrant background and native Dutch origin. Qualitative data contribute insight into the individual experience of socially excluded people and can be used to explore whether items sufficiently represent the same content across cultures [32].

## Materials and methods

### Mixed methods design

**Fig 1:**
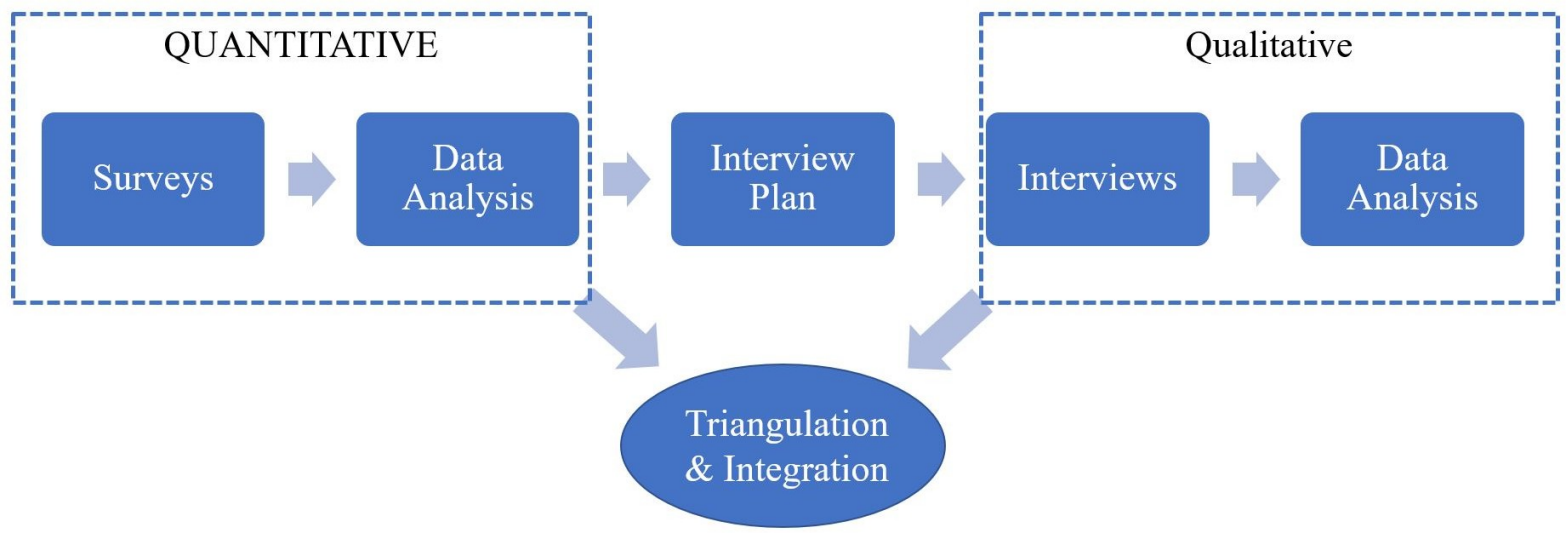
Flowchart study design.

The present study has a sequential explanatory mixed methods design consisting of a dominant quantitative and a less dominant qualitative phase [33, 34]. Fig 1 shows the sequence, priority and integration of the two phases. In phase I survey data were collected on SE in the general population. In phase II, data from phase I were used to select a sample of socially excluded persons of Surinamese, Moroccan, Turkish and native Dutch origin. Semi-structured interviews were conducted on the perspective of the respondents on their situation and responses on the SEI-HS. The Medical Ethics Review Committee of the AMC confirmed that under Dutch law, medical ethics approval was not required for phase I (AMC, W12_146 no. 12.17.0163) nor for phase II (AMC, W13_311 # 14.17.0007) as participants were not subjected to any intervention or treatment.

### I Quantitative phase

#### Data collection

The quantitative data were collected by the Public Health Services of the four largest cities in the Netherlands, as part of the Public Health Monitor (PHM) 2012. The PHM is a nationwide self-report survey of non-institutionalised adults aged 19 years or older, conducted every four years. To ensure that elderly and people living in neighbourhoods with a low socioeconomic status were well represented, stratified samples were drawn by Statistics Netherlands, based on age and neighbourhood. In total 71,627 residents of Amsterdam, Rotterdam, The Hague and Utrecht were invited to participate. Non-responders received two written reminders, and in the case of non-western immigrants, an extra telephone call or home visit. Questionnaires in Turkish, Moroccan Arabic and English translation could be used and trained interviewers were available to assist respondents face-to-face or by phone in their preferred language (Dutch, Arabic, Berber, Turkish or English). The response rate was 40% (Surinamese 28%, Moroccan 26%, Turkish 26%, Dutch 48%). Statistics Netherlands enriched the PHM data with information on zip code, migration background and standardised household income [35]. Participation in the research was anonymous and voluntary. In accordance with the Dutch Law, participants were informed by letter that by completing the questionnaire they consent with anonymous use of data for research.

#### Measurement

Social exclusion. The SEI-HS consists of 17 items which measure four dimensions and an overall index of SE [24]. The four dimensions are: 1) lack of social participation, 2) material deprivation, 3) inadequate access to basic social rights and 4) lack of normative integration. Scores on the index and the four dimensions are categorised into ‘little or no’, ‘some’ and ‘moderate to strong’ exclusion. The SEI-HS was validated in the general Dutch population. The items were derived from various validated questionnaires such as the Loneliness scale of De Jong Gierveld [36], the SCP Social Exclusion Index [20] and Social Cohesion and Trust [22]. The internal consistency, internal structure, construct validity and generalisability were found satisfactory [24].

Migration background. In line with the Dutch standard definition, country of birth or, in case of second-generation immigrants, country of birth of the mother and/or father, as registered in the municipal population registers, were used to define migration background.

#### Quantitative data analysis

Descriptive statistics. Analyses were restricted to respondents of Surinamese, Moroccan and Turkish origin with native Dutch respondents as the reference group. In order to control for the stratified sampling design and selective non-response, we used SPSS Version 22 Complex Samples Likelihood tests for the descriptive analyses of the prevalence of SE. Sampling weights were calculated by Statistics Netherlands based on a linear model with 9 sociodemographic variables and their interaction terms [37]. The significance level α was set at 0.001 to reflect the large sample size.

Structural validity. To test whether the SEI-HS factor structure holds across the three migrant groups, we conducted confirmatory factor analyses in data subsets per migrant group using SPSS Amos 22.0. Five of the standard goodness-of-fit statistics given in Amos were used to assess model fit i.e. root mean square error of approximation (RMSEA), upper bound of 90% confidence interval (HI90), Tucker-Lewis index (TLI), comparative fit index (CFI) and Hoelter’s .05 Index [38]. The Chi square statistic was not considered given its sensitivity to large sample sizes. The model fit was considered good if RMSEA< 0.05, HI90) < 0.06, TLI ≥ 0.95, CFI > 0.90 and Hoelter’s .05 Index ≥ 200 [38]. These same criteria were used in the development of the SEI-HS [24].

Differential item functioning (DIF). DIF occurs when one group of individuals responds differently from another group on a given questionnaire item, even though both groups are equivalent on the underlying construct that is assessed, or in DIF terminology, if both groups show the same ability on the matching variable. In this study the categories ‘little or no’, ‘some’ and ‘moderate to strong’ of the relevant dimension scale were used as the ability levels. The cut-off points for these categories were based on the 85^th^ and 95^th^ percentile in the Dutch adult population of 19 years or older in 2012 [24]. For each immigrant group, three hierarchical models were calculated with SPSS ordinal logistic regression, with Y being the SEI-HS item tested, M the matching variable (i.e. the corresponding SE dimension) and G the grouping variable (i.e. Surinamese, Moroccan or Turkish versus Dutch):

Model 1: *Y* = *β*_0_+*β*_1_*M*;

Model 2: *Y* = *β*_0_+*β*_1_*M*+*β*_2_*G*;

Model 3: *Y* = *β*_0_+*β*_1_*M*+*β*_2_*G*+ *β*_3_M*G;

An item was considered to exhibit substantial DIF if the difference between model 1 and 3 in log-likelihoods was statistically significant (α = 0.001) and the change in R^2^ at least moderate according to the Jodoin-Gierl effect size criteria by which ΔR2 < 0.035 is classified as negligible; 0.035 ≤ ΔR2 ≤ 0.070 as moderate and ΔR2>0.070 as large. [39–41]. In case of substantial DIF further analyses were made to characterize the type of DIF into uniform DIF (significant difference between model 1 and 2) and/or non-uniform DIF (significant difference between model 2 and 3). Criteria can be found in S1A–S1C Table.

### II Qualitative phase

In the qualitative part of the study we set out to describe, analyse and compare the experiences of social exclusion and the responses on the SEI-HS in the four research groups. We followed the consolidated criteria for reporting qualitative research (COREQ) checklist [42].

#### Participant selection

The sampling frame consisted of the respondents of Surinamese, Moroccan, Turkish and Dutch background, with a high score on the SEI-HS who had given the Public Health Services written consent to re-contact (Table 1). Respondents from the city of Rotterdam could not be included as permission had not been requested. To reflect the variability in gender, age and neighbourhood across the four research groups, in total 50 cases were selected at random from the different strata. In case of non-response a replacement was selected as similar as possible to the original case.

**Table 1:** Number of respondents qualitative and quantitative phase.

	Quantitative survey	Qualitative interview			
	Phase I Response (%)	High score on SEI-HS	Agreed to follow-up *	Phase II Sample	Phase II Response (%)
Surinamese	1,803 (28%)	277	101	27	11 (41%)
Moroccan	1,009 (26%)	174	72	43	9 (21%)
Turkish	1,164 (26%)	235	72	43	10 (23%)
Dutch	19,318 (48%)	277	71	64	22 (34%)
	23.294 (42%)	879	316	177	52 (29%)

* Follow-up from Amsterdam, The Hague and Utrecht.

#### Data collection

Interviews took place between March and September 2014. During this period 177 respondents were contacted by letter, telephone and home visits. Up to three attempts were made to get in touch. The response rates are shown in Table 1, with no contact being the main reason for non-response (not at home or moved house).

Interviews took place at a time and location convenient to the respondent, generally at their home address. Signed informed consent was obtained at the time of the interview. Each respondent received a 20 euro gift card as compensation for their time. The interviews were conducted by two experienced members of the research team (CB, AvL), of Dutch and Indonesian background respectively, and students of Surinamese, Moroccan and Turkish background. Students were trained by members of the research team and closely supervised in their work. The supervision not only focused on methodological aspects but also on emotional wellbeing and safety of the students.

To explore the perceptions of the respondents, a semi structured topic guide was used which comprised open-ended questions accompanied by probes and prompts to expand, clarify and understand responses. The 17 items of the SEI-HS were asked exactly as worded, but further explanation was given if the respondent asked for it. Other topics included health and health behaviour, feelings of being left out of society, locus of control and expectations for the future. To create a pleasant and personal atmosphere, respondents were invited, at the start of the interview, to tell something about themselves and the things they enjoy doing. Interviews lasted 20–90 minutes (53 minutes on average), depending on the willingness and ability of the respondents. Interviews were audio-recorded and transcribed verbatim by independent transcriptionists.

#### Qualitative data analyses

The transcribed interviews were entered in MaxQDA and analysed by two research team members (BC, AvB) using thematic coding techniques. The initial coding framework was based on the structure of the topic guide. Subsequently, for each SEI-HS item text references were analysed on semantic, conceptual and contextual evidence and categorised [32]. Semantic evidence included all text references referring to the meaning of the language used and the comprehensibility of the item. The text references were coded ‘0’ if respondents correctly understood the wording of the item, ‘1’ if that was not the case and ‘x’ if there was no conclusive evidence. Conceptual evidence included all text references referring to the general idea or notion captured by the item. The conceptual connotations were compared with the intended concept of the item and coded as either equivalent (0), deviating (1) or inconclusive (x). Contextual evidence included all text referring to the contextual specificity of items. This specificity only becomes apparent through between-group comparison [32].The text references were coded per respondent as: ‘0’ if no culturally specific context was mentioned or appeared to play a role in the respondents answer, ‘1’ if culturally specific context was mentioned and ‘x’ if there was no conclusive evidence.

Scores were calculated for each research group and each type of evidence. If 30% or more of the responses was problematic i.e. coded ‘1’, we categorised this as ‘yes, there may be a reason for concern’; if 10–30% was problematic, we categorised this as ‘perhaps, there is a reason for concern; and 0–10% was categorised as ‘no reason for concern’. Cases with inconclusive evidence were excluded from the calculation.

Finally, all responses coded ‘yes, there may be a reason for concern’ were compared between the groups and analysed for their potential effect on the cross-cultural validity.

Reporting in this manuscript follows the STROBE guidelines for cross-sectional studies [43].

## Results

### I Quantitative phase

#### Descriptive statistics

Background characteristics. Table 2 shows that the Dutch respondents of phase 1 are generally older than the three immigrant groups and live less often in neighbourhoods with a low socioeconomic status (SES).

**Table 2:** General characteristics of respondents by migration background, Phase I and II (%).

	Women	19–39 years	40–64 years	65 years and older	Low SES neighbourhood	N
PHASE I: Quantitative survey						
Surinamese	59.1	30.1	37.4	32.6	49.3	1,803
Moroccan	50.4	41.1	40.1	18.7	60.8	1,009
Turkish	52.0	46.1	36.9	17.0	66.3	1,164
Dutch	55.2	28.9	28.1	43.0	26.2	19,318
PHASE 2: Qualitative interview						
Surinamese	63.6	36.4	36.4	27.3	54.5	11
Moroccan	44.4	33.3	55.6	11.1	77.8	9
Turkish	50.0	20.0	80.0	0.0	70.0	10
Dutch	50.0	18.2	40.9	40.9	59.6	22

Social exclusion. The data presented in Table 3 confirm that in the four cities SE is more prevalent in adults of Surinamese, Moroccan and Turkish origin compared to native Dutch adults. High levels of SE were found in 20.0% of the urban population of Surinamese origin, 20.9% of the Moroccan, 28.7% of Turkish and 4.2% of native Dutch origin. Elevated levels were also found on the underlying dimension scales. Especially material deprivation was increased in all three immigrant groups by a factor of 6 to 7. Inadequate access to basic social rights was highest in adults of Moroccan origin. Only in Turkish adults, the prevalence of ‘Lack of normative integration’ was not increased compared to adults of native Dutch origin (p = 0.023).

**Table 3:** Prevalence rates of moderate to strong social exclusion in adults of Surinamese, Moroccan, Turkish and Dutch origin#.

	Surinamese (N = 1,803)		Moroccan (N = 1,009)		Turkish (N = 1,164)		Dutch (N = 19,318)
	%	p	%	p	%	p	%
SEI-HS index	20.0	.*000*	20.9	.*000*	28.7	.*000*	4.2
Dim1: limited social participation	13.4	.*000*	11.6	.*000*	17.2	.*000*	4.4
Dim 2: material deprivation	24.1	.*000*	22.6	.*000*	25.2	.*000*	3.6
Dim 3: inadequate access to basic social rights	16.5	.*000*	27.2	.*000*	22.7	.*000*	5.3
Dim 4: lack of normative integration	15.7	.*000*	12.4	.*000*	9.5	.023	6.4

# Prevalence rates were weighted for sample design and selective non-response. SPSS Complex Samples Likelihood-test was used to test the difference with the Dutch reference group. P-value italic if significant at < 0.001 level.

#### Confirmatory factor analyses

The results showed an acceptable model fit for the three immigrant groups (Table 4). In all cases the Hoelter’s .05 Index indicated good model fit. Factor loadings were all significant at the 0.001 level except for item 17 ‘Work is just a way of earning money’ (Table 4). The factor loadings of this item were not significant in the Moroccan and Turkish groups. The RMSEA, CFI and TLI coefficients were comparable to the fit of the original SEI-HS model.

**Table 4:** Confirmatory Factor Analysis of the SEI-HS in adults of Surinamese, Moroccan and Turkish origin compared with prior validation results in the Dutch adult population [24].

	RMSEA (< 0.050)	HI90 (< 0.060)	TLI (≥ 0.950)	CFI (> 0.900)	Hoelter’s .05 Index (≥ 200)	Factor loadings significant (p<0.001)
Surinamese	0.056	0.060	0.846	0.887	***334***	All items
Moroccan	0.063	0.069	0.781	0.838	***246***	All but Item#17 (p = 0.052) ^a^
Turkish	0.058	0.062	0.839	0.881	***320***	All but Item#17 (p = 0.299) ^a^
Validation in Dutch adult population [24]	0.057	***0*.*058***	0.827	0.872	***407***	All items

RMSEA = root mean square error of approximation; HI90 = upper bound of 90% confidence interval; TLI = Tucker-Lewis index; CFI = comparative fit index. Results in italic and bold if RMSEA < 0.05, HI90 < 0.06, TLI ≥ 0.95, CFI > 0.90 and Hoelter’s .05 Index ≥ 200, indicating good model fit.

^a^ Item 17: Work is just a way of earning money. For more details see S2 Table.

#### Differential item functioning

Of the 17 items examined, none displayed substantial DIF i.e. p < 0.001 and ΔR^2^ 0.035 or higher (S1A–S1C Table).

### II Qualitative phase

In total 52 interviews were conducted, with respectively 11 Surinamese, 9 Moroccan, 10 Turkish and 22 Dutch persons. Four in five were interviewed by an interviewer of the same migration background (81%). Characteristics of respondents are presented in Table 2.

For each SEI-HS item the semantic, conceptual and contextual connotations reported by the respondents were coded and compared between the four research groups. As can be seen from Table 5 the items of dimension 4 caused most reason for concern. Semantic problems were identified for all groups (including native Dutch respondents) in item 17. The item was misunderstood by more than a third of the respondents (12 out of 33). Instead of ‘working is just a way of earning money’ most of them understood the item as ‘working is an unjust way of earning money’. Coincidentally, a negative answer indicates in both cases normative integration and a positive answer the lack thereof. Semantic problems with item 15 (I sometimes do something for my neighbours) concerned primarily Moroccan respondents.

**Table 5:** Qualitative findings on content-related validity by migration background: Extent of reason for concern *.

	Semantic evidence				Conceptual evidence				Contextual evidence			
	Surin.	Moroc.	Turkish	Dutch	Surin.	Moroc.	Turkish	Dutch	Surin.	Moroc.	Turkish	Dutch
**Dimension 1: Limited social participation**												
1. I experience a general sense of emptiness^1^	x 2/3	x 1/2	yes 2/6	no 1/13	perhaps 1/6	x 0/2	x 0/4	no 0/13	x 1/2	x 0/4	perhaps 2/7	no 1/13
2. There is always someone I can talk to about my day-to-day problems^1^	no 0/5	x 1/3	x 1/3	no 1/11	no 0/5	x 0/4	x 1/3	perhaps 3/11	no 0/5	x 3/4	x 1/3	no 0/11
3. There are plenty of people I can lean on when I have problems^1^	x 0/3	x 2/3	x 1/4	no 1/11	no 0/6	perhaps 1/5	perhaps 1/6	no 1/11	no 0/6	no 0/5	no 0/6	no 0/11
4. I miss the pleasure of the company of others^1^	perhaps 1/7	x 1/4	x 0/3	no 0/8	no 0/7	no 0/5	x 0/3	no 0/9	no 0/7	no 0/5	x 0/3	no 0/9
5. I often feel rejected^1^	perhaps 1/7	perhaps 1/6	perhaps 1/7	no 0/19	perhaps 2/7	x 0/4	perhaps 1/8	perhaps 3/18	no 0/7	x 1/3	yes 3/8	no 0/17
6. Little contact with neighbours and people in the street^2^	x 0/3	x 0/3	no 0/5	no 0/13	x 0/3	no 0/5	no 0/5	no 0/13	x 0/3	yes 3/5	yes 4/5	no 1/13
**Dimension 2: material deprivation**												
7. Had difficulty past year getting by on the household income^2^	no 0/5	x 1/3	no 0/5	no 0/18	no 0/10	no 0/9	no 0/10	no 0/22	no 0/10	no 0/9	no 0/10	no 0/22
8. I have enough money to heat my home^3^	yes 3/7	x 0/4	no 0/7	no 0/16	no 0/6	no 0/6	no 0/8	no 0/14	no 0/6	no 0/6	no 0/8	no 0/14
9. I have enough money for club memberships^3^	perhaps 1/6	x 1/3	perhaps 2/8	no 1/17	no 0/8	no 0/5	no 0/8	no 0/20	no 0/8	no 0/5	no 0/8	no 0/20
10 I have enough money to visit others^3^	no 0/5	x 1/4	no 0/7	no 0/14	no 0/10	no 0/9	no 0/10	no 0/19	yes 4/10	yes 4/9	yes 8/10	perhaps 2/19
**Dimension 3: inadequate access to basic social rights**												
11 People in this neighbourhood generally do not get along with each other^4^	perhaps 1/9	x 0/4	no 0/6	no 0/15	no 0/9	no 0/8	no 0/8	no 0/15	no 0/9	no 0/8	no 0/8	no 0/15
12 Degree of satisfaction with housing^2^	no 0/11	yes 5/9	perhaps 2/9	no 0/22	no 0/7	no 0/8	no 0/8	no 0/22	no 0/7	no 0/8	no 0/8	no 0/22
13 I didn’t receive a medical or dental treatment^3^	no	perhaps	perhaps	no	no	no	no	no	no	no	no	no
	0/9	2/7	1/6	1/17	0/8	0/18	0/9	0/8	0/8	0/18	0/9	0/8
**Dimension 4: lack of normative integration**												
14 I give to good causes^3^	perhaps 1/8	perhaps 1/5	no 0/5	no 1/15	yes 6/8	perhaps 1/5	yes 3/7	yes 7/15	no 0/8	yes 2/5	perhaps 1/7	no 0/15
15 I sometimes do something for my neighbours^3^	perhaps 1/8	yes 3/6	no 0/5	no 0/15	yes 3/8	yes 2/6	yes 3/7	yes 5/17	no 0/8	perhaps 1/6	perhaps 1/7	no 1/16
16 I put glass items in the glass recycling bin^3^	no 0/8	perhaps 2/7	no 0/5	no 1/15	perhaps 1/9	perhaps 1/8	no 0/7	perhaps 2/15	no 0/9	perhaps 1/8	no 0/7	no 0/15
17 Work is just a way of earning money^3^	yes 2/6	yes 2/5	yes 3/5	perhaps 5/17	no 0/6	perhaps 1/5	perhaps 2/7	perhaps 4/17	no 0/5	x 0/2	no 0/6	no 0/16

Legend: no = no reason for concern i.e. 0–10% of the respondents did not understand the wording or formulation (semantic evidence), reported a different connotation than intended (conceptual evidence) or mentioned culturally specific context (contextuel evidence). x = insufficient information (less than 5 observations); perhaps = perhaps, there is some reason for concern: 10–30% of the respondents met the above criterion; and yes = yes, there may be a reason for concern: > = 30% met the criterion.

Cell colour: yellow = potential threat to the cross-cultural valdity; green = no threat tot the cross-cultural validity; blue = general validity issue.

* The Dutch version of the SEI-HS can be found in S1 Appendix.

^1^ Loneliness scale De Jong & Gierveld [23].

^2^ Dutch Public Health Monitor [21].

^3^ SCP Social excusion index [20].

^4^ Social Cohesion and Trust scale [22].

Items 14, 15 and 17 of dimension 4 showed conceptual problems in all four groups. Item 14 measured in almost half of the respondents (15 out of 32) lack of money instead of noncompliance to the core values of Dutch society: “*I have a few charities that are my favourites*, *they really need it*. *But my finances are at a pretty low ebb at the moment*.” Item 15 measured in one third of the respondents (18 out of 37) lack of opportunity to do something for your neighbours (e.g. in case of conflict or no contact with neighbours) and/or inability to help (e.g. due to old age or ill health). Item 17 measured in one fifth of the respondents (7 out of 35) work ethic instead of noncompliance to core values. These respondents found work a good way to earn money: “*If you don't work*, *you won't eat*”. Contextuality played a role in item 14. One Moroccan and one Turkish respondent mentioned payment to the mosque. This works both ways: “*If they come from the mosque*, *I pretend I don't hear anything*, *they think 2 or 3 euros is too little*.” One Moroccan respondent paid medical costs for poor family members in the home country.

The items of dimension 2 and 3, ‘Material deprivation’ and ‘Access to basic social rights’, gave less reason for concern. A number of respondents had difficulty in understanding the wording of the items 8 and 12. Three Surinamese respondents (3 out of 7) did not answer item 8 if they have enough money to heat the house properly, but whether the house can be heated well: “*I hope so*, *I have not experienced the winter here yet*”. Five Moroccan respondents (5 out of 9) were not able to translate their (dis)satisfaction with their home (item 12) into a corresponding grade. Our analysis did not suggest any conceptual problems: all respondents interpreted the items of dimension 2 and 3 as intended. Contextuality only played a role in item 10. Having enough money to visit others did not only depend on the financial situation of the household but also on the travel costs incurred. Family of immigrants generally live further away, making travel costs more difficult to pay.

The items of dimension 1 also functioned much as expected, with some exceptions. Item 1 was not understood by a quarter of the respondents (6 out of 24), both immigrants and one native Dutch respondent: “*Emptiness*? *What do you mean by that*?”. Item 5 showed comparatively the most validity problems. Six respondents, both immigrants (3 out of 17) and native Dutch (3 out of 18), reported that they felt rejected by their employer or by institutions like the tax office or the Employee Insurance Agency. Conceptually this interpretation belongs more to dimension 3 ‘Access to institutions’ than to ‘Social Participation’. In four cases the events or cases referred to were specific to the cultural group, for example forced marriage in case of a Turkish respondent. Contextuality also plays a role in item 6. The degree of contact that Moroccan, Turkish and Dutch respondents have with their neighbours is influenced by the migration background of these neighbours. According to a Turkish respondent they just say “*hi*” to the Dutch neighbours, but visit their Turkish neighbours regularly at home. The concept that is being measured, however, does not differ between the groups.

## Discussion

Our objective was to examine possible shortcomings in the cross-cultural validity of the SEI-HS that might explain the high prevalence of SE in adult immigrant groups found in the 2012 health monitor. The study was conducted among adults of Surinamese, Moroccan, Turkish and Dutch origin in the four largest cities in the Netherlands. The quantitative part of the study showed no cross-cultural validity issues. CFA confirmed the 4-factor structure of the SEI-HS in the three immigrant groups and none of the SEI-HS items exhibited problems with differential item functioning. Item scores did not differ significantly between respondents of Surinamese, Moroccan, Turkish origin and native Dutch respondents at the same level of SE. The qualitative part uncovered little differences in understanding and interpretation of items between the population groups, but some general methodological shortcomings were identified, especially in the normative integration dimension of the SEI-HS.

The socially excluded respondents we interviewed did not always interpret the items as intended, due to unfamiliarity with words, complicated sentence structures and different connotations. Potential cultural biases were limited to the semantics of items 8,12 and 15 and contextuality of items 5 and 10. The interviews showed that particularly Moroccan respondents had problems understanding certain items. Rewording or rephrasing of semantically difficult items could be considered. In general, these findings underline the importance of offering assistance to respondents face-to-face or by phone in their own language (Berber or Arabic). Items 5 (I often feel rejected) and 10 (I have enough money to visit others) showed contextual differences that might threaten the cultural validity of the items. This was however not reflected in the quantitative analyses.

Most validity issues were as noteworthy in native Dutch respondents as in Surinamese, Moroccan and Turkish respondents. This was not expected since all SEI-HS items originate from widely used and/or validated questionnaires [20–23]. The content of items 8–10 and 13–17 was derived from literature and interviews, judged by four focus groups and tested through individual cognitive interviews [20]. Efforts were made to include people with a higher risk of SE i.e. with low income and low educational level. The content of items 1–5 was derived from literature, life histories and interviews and judged by researchers and students [44]. Item 11 stems from a validated scale [45] that was translated into Dutch with back translation into English [22]. As far as we could establish, these items were not pre-tested among persons from disadvantaged social groups and/or low education or income.

Despite the fact that the Normative Integration items were pretested with low-income and low-education participants, several issues with semantic and conceptual validity were encountered. The concept of normative integration touches on the moral underclass discourse, one of three models of social exclusion identified by Levitas [46]. The discourse focuses on the behavioural and attitudinal characteristics of the excluded and their imputed deficiencies. The Normative Integration scale developed by the SCP [20] reflects a fairly narrow spectrum of behaviours and attitudes that are relatively common in the general Dutch population. Our study showed that high scores on lack of normative integration do not necessarily reflect a lack of social commitment or anomie, but may reflect an inability to comply. For example, not helping your neighbours because you are handicapped yourself or not donating to good causes because you are in serious debt. One could argue that concept and social group are coming together here and that the failure to comply with given norms and values is part and parcel of the exclusion itself. From this point of view, the validity of the Normative Integration scale need not be jeopardised. High scores on the Normative Integration scale reflect high social exclusion, even though the interpretation of the concept and context may differ between respondents. Further research in the non-excluded group could shed more light on this issue.

A strong point of our study is the use of a sequential explanatory mixed methods design for validation purposes. This approach is not very common. Usually, qualitative research precedes quantitative validation and not vice versa [47]. Although uncommon, the approach has been used before. For example, Morren et al.[48] interviewed respondents with deviant response style behaviour and Carlier et al.[49] approached groups with high levels of non-response. In our case, the design allowed us to address reliability and validity issues that were uncovered in the quantitative survey. It also allowed to confirm the ability of the social exclusion index to identify a diverse group of socially excluded persons including perpetrators of domestic violence, persons leading very isolated lives, victims of violent incidents such as armed robbery or rape, people with drug addiction or aggression disorder, and someone just released from detention.

There are some limitations to our study. The first limitation is related to the low response rate of the PHM especially among non-western immigrant groups. Although the Public Health Services employed a large range of measures to increase participation of difficult to reach groups, a certain degree of selection bias e.g. for better integrated and educated immigrants, is inevitable. The great diversity within the qualitative research group gave us, however, confidence in the representativeness of the research outcomes. Another limitation is that the research was conducted only in urban areas. Lastly, we classified the persons in our research based on their country of birth and that of their parents. This classification does not necessarily define their individual identity or represent meaningful social categories [50]. Gender, age, occupation, ethnic identity and educational level, may be more relevant in certain contexts than migration background. As more detailed knowledge becomes available, it becomes more difficult to make statements about immigrant groups in general [51].

## Conclusions

The results of this study support the cross-cultural validity of the SEI-HS in three major non-western immigrant groups in the Netherlands. The findings suggest that the large differences in SE found between native Dutch and non-western immigrant groups are real and not due to measurement bias. This raises serious concerns about the social inclusion of non-western immigrants in the four largest cities in the Netherlands and its potential effect on health and wellbeing. Policy measures to reduce SE are urgently needed as well as more research into the mechanisms and risk factors of SE among immigrant groups and pathways to more social inclusion.

Further research is necessary to examine the content validity of the normative integration dimension of the SEI-HS and rephrasing semantically problematic items. The interviews showed that the lived experience of socially excluded people may differ from the majority population. In general, it is advisable to involve people in adverse social circumstances in the development of health related measures.

## Supporting information

S1 Table (A-C) Differential item functioning in SEI-HS items with respect to migrant background, A: Surinamese, B: Moroccan and C Turkish versus native Dutch.
(PDF)

S2 Table Factor loadings items SEI-HS in adults of Surinamese, Moroccan and Turkish origin compared to the reference values in the general Dutch population. (PDF)

S1 Appendix Dutch version of the SEI-HS. (PDF)
