# Supplementary material for: Evaluating the cross-cultural validity of the Dutch version of the Social Exclusion Index for Health Surveys (SEI-HS): A mixed methods study
Source: PLoS One. 2019 Nov 5;14(11):e0224687. doi: 10.1371/journal.pone.0224687 (PMC6830809; doi:10.1371/journal.pone.0224687)
Supplement: S1 Table — (A-C) Differential item functioning in SEI-HS items with respect to migrant background, A: Surinamese, B: Moroccan and C Turkish versus native Dutch. (PDF) [file pone.0224687.s001.pdf]

S1A Table. Differential item functioning in SEI-HS items with respect to migrant background, Surinamese versus native Dutch. * **

Surinamese versus native Dutch	Total DIF		Uniform DIF				Non-uniform DIF		Type of DIF
	P Value M3 vs M1	Δ R^2 M3-M1	*P* Value M2 vs M1	% difference in β_{11} (M2-M1)	% difference in β_{12} (M2-M1)	Δ R^2 M2-M1	*P* Value M3 vs M2	Δ R^2 M3-M2	***
**Dimension 1: Limited social participation**									
1. I experience a general sense of emptiness	0.003	0.003	0.003	-1.4%	-0.9%	0.002	0.031	0.001	None
2. There is always someone I can talk to about my day-to-day problems	**0.000**	0.007	**0.000**	-3.1%	-2.2%	0.006	0.050	0.001	Not subst.
3. There are plenty of people I can lean on when I have problems	**0.002**	0.003	**0.000**	-1.9%	-1.5%	0.002	0.573	0.001	Not subst.
4. I miss the pleasure of the company of others	0.016	0.002	0.019	-1.2%	-0.8%	0.001	0.087	0.001	None
5. I often feel rejected	0.065	0.001	0.028	-1.1%	-0.7%	0.001	0.299	0.000	None
6. Little contact with neighbours and people in the street	0.162	0.001	0.412	-0.5%	-0.4%	0.000	0.107	0.001	None
**Dimension 2: material deprivation**									
7. Had difficulty past year getting by on the household income	**0.000**	0.008	**0.000**	-3.2%	-1.9%	0.004	**0.000**	0.004	Not subst.
8. I have enough money to heat my home	**0.002**	0.006	**0.000**	-4.7%	-0.9%	0.005	0.266	0.001	Not subst.
9. I have enough money for club memberships	**0.000**	0.004	**0.000**	-4.0%	-2.8%	0.005	**0.000**	0.005	Not subst.
10 I have enough money to visit others	0.019	0.002	0.005	-2.1%	-2.0%	0.002	0.375	0.000	None
**Dimension 3: inadequate access to basic social rights**									
11 People in this neighbourhood generally do not get along with each other	**0.000**	0.010	**0.000**	-2.8%	-2.3%	0.009	0.023	0.001	Not subst.
12 Degree of satisfaction with housing	0.993	0.000	0.971	0.0%	0.0%	0.000	0.955	0.000	None
13 I didn't receive a medical or dental treatment	0.035	0.004	0.025	-2.7%	-2.6%	0.002	0.164	0.002	None
**Dimension 4: lack of normative integration**									
14 I give to good causes	**0.001**	0.001	0.550	0.3%	0.3%	0.000	**0.000**	0.001	Not subst.
15 I sometimes do something for my neighbours	**0.000**	0.011	**0.000**	-2.5%	-2.2%	0.010	0.172	0.001	Not subst.
16 I put glass items in the glass recycling bin	**0.000**	0.005	**0.001**	-1.7%	-1.9%	0.002	**0.000**	0.003	Not subst.
17 Work is just a way of earning money	**0.000**	0.009	**0.000**	-3.2%	-7.3%	0.008	0.004	0.001	Not subst.

* Model 1: $Y = \beta_0 + \beta_1 M$; Model 2: $Y = \beta_0 + \beta_1 M + \beta_2 G$; Model 3 : $Y = \beta_0 + \beta_1 M + \beta_2 G + \beta_3 M*G$. Y=item, M=matching variabele=dimensions scale en G=grouping variable=Surinames vs native Dutch.

** Results in bold if the following criteria for DIF were met: P value < 0.001, % difference in β > 10% [39], Nagelkerke pseudo R^2 Δ >= 0.035 [40].

*** None: P value total DIF >= 0.001; Not substantial: P value total DIF < 0.001 & Δ R^2 (M3-M1) < 0.035.

S1B Table. Differential item functioning in SEI-HS items with respect to migrant background, Moroccan versus native Dutch. * **

Moroccan versus native Dutch	Total DIF		Uniform DIF				Non-uniform DIF		Type of DIF
	P Value M3 vs M1	Δ R^2 M3-M1	*P* Value M2 vs M1	% difference in β_{11} (M2-M1)	% difference in β_{12} (M2-M1)	Δ R^2 M2-M1	*P* Value M3 vs M2	Δ R^2 M3-M2	***
**Dimension 1: Limited social participation**									
1. I experience a general sense of emptiness	0.009	0.002	0.235	-0.7%	-0.4%	0.000	0.006	0.002	None
2. There is always someone I can talk to about my day-to-day problems	**0.000**	0.005	**0.000**	-2.6%	-1.6%	0.005	0.628	0.000	Not subst.
3. There are plenty of people I can lean on when I have problems	**0.000**	-0.001	**0.000**	-0.5%	-2.2%	-0.002	0.179	0.001	Not subst.
4. I miss the pleasure of the company of others	**0.000**	0.004	**0.000**	-1.8%	-1.0%	0.003	0.030	0.001	Not subst.
5. I often feel rejected	0.445	0.000	0.685	0.2%	0.1%	0.000	0.286	0.000	None
6. Little contact with neighbours and people in the street	0.340	0.001	0.086	1.1%	1.1%	0.001	0.816	0.000	None
**Dimension 2: material deprivation**									
7. Had difficulty past year getting by on the household income	**0.000**	0.003	0.026	-1.3%	-1.1%	0.001	**0.000**	0.002	Not subst.
8. I have enough money to heat my home	**0.000**	0.024	**0.000**	-7.2%	-8.4%	0.023	0.307	0.001	Not subst.
9. I have enough money for club memberships	**0.000**	0.026	**0.000**	-4.3%	-3.2%	0.021	**0.000**	0.005	Not subst.
10 I have enough money to visit others	1.000	0.000	1.000	0.0%	0.3%	0.000	1.000	0.000	None
**Dimension 3: inadequate access to basic social rights**									
11 People in this neighbourhood generally do not get along with each other	**0.000**	0.008	0.110	1.4%	0.9%	0.000	**0.000**	0.008	Not subst.
12 Degree of satisfaction with housing	**0.000**	0.011	**0.000**	-3.1%	-2.6%	0.008	**0.000**	0.003	Not subst.
13 I didn't receive a medical or dental treatment	0.346	0.002	0.560	-1.2%	-0.9%	0.000	0.227	0.002	None
**Dimension 4: lack of normative integration**									
14 I give to good causes	0.695	0.000	0.308	0.4%	0.3%	0.000	0.815	0.000	None
15 I sometimes do something for my neighbours	0.411	0.000	0.404	-0.3%	-0.2%	0.000	0.336	0.000	None
16 I put glass items in the glass recycling bin	**0.000**	0.017	**0.000**	-2.4%	-2.1%	0.015	0.004	0.002	Not subst.
17 Work is just a way of earning money	**0.000**	0.005	0.078	-0.7%	-0.9%	0.000	**0.000**	0.005	Not subst.

* Model 1: $Y = \beta_0 + \beta_1 M$; Model 2: $Y = \beta_0 + \beta_1 M + \beta_2 G$; Model 3 : $Y = \beta_0 + \beta_1 M + \beta_2 G + \beta_3 M*G$. Y=item, M=matching variabele=dimensions scale en G=grouping variable=Moroccan vs native Dutch.

** Results in bold if the following criteria for DIF were met: P value < 0.001, % difference in β > 10% [39], Nagelkerke pseudo R^2 Δ >= 0.035 [40].

*** None: P value total DIF >= 0.001; Not substantial: P value total DIF < 0.001 & Δ R^2 (M3-M1) < 0.035.

S1C Table. Differential item functioning in SEI-HS items with respect to migrant background, Turkish versus native Dutch. * **

Turkish versus native Dutch	Total DIF		Uniform DIF				Non-uniform DIF		Type of DIF
	P Value M3 vs M1	Δ R^2 M3-M1	*P* Value M2 vs M1	% difference in $\beta_{11\ (M2\text{-}M1)}$	% difference in $\beta_{12\ (M2\text{-}M1)}$	Δ R^2 M2-M1	*P* Value M3 vs M2	Δ R^2 M3-M2	***
**Dimension 1: Limited social participation**									
1. I experience a general sense of emptiness	**0.000**	0.016	**0.000**	-4.2%	-3.1%	0.016	0.079	0.000	Not subst.
2. There is always someone I can talk to about my day-to-day problems	**0.000**	0.004	**0.000**	-3.4%	-2.6%	0.003	0.234	0.001	Not subst.
3. There are plenty of people I can lean on when I have problems	**0.000**	0.009	**0.000**	-4.2%	-3.3%	0.006	**0.001**	0.003	Not subst.
4. I miss the pleasure of the company of others	**0.000**	0.017	**0.000**	-4.4%	-3.4%	0.017	0.583	0.000	Not subst.
5. I often feel rejected	**0.001**	0.003	**0.000**	-2.1%	-1.6%	0.003	0.790	0.000	Not subst.
6. Little contact with neighbours and people in the street	**0.000**	0.003	0.000	4.0%	4.5%	0.003	0.103	0.000	Not subst.
**Dimension 2: material deprivation**									
7. Had difficulty past year getting by on the household income	**0.000**	0.006	**0.000**	-3.5%	-3.3%	0.006	0.182	0.000	Not subst.
8. I have enough money to heat my home	**0.000**	0.014	**0.000**	-6.1%	-8.0%	0.013	0.274	0.001	Not subst.
9. I have enough money for club memberships	**0.000**	0.021	**0.000**	-4.7%	-4.6%	0.017	**0.000**	0.004	Not subst.
10 I have enough money to visit others	1.000	0.000	1.000	-0.9%	4.6%	0.000	1.000	0.000	None
**Dimension 3: inadequate access to basic social rights**									
11 People in this neighbourhood generally do not get along with each other	**0.000**	0.003	0.033	-1.5%	-0.9%	0.000	**0.000**	0.003	Not subst.
12 Degree of satisfaction with housing	**0.000**	0.002	0.641	0.2%	0.2%	0.000	**0.000**	0.002	Not subst.
13 I didn't receive a medical or dental treatment	**0.000**	0.030	**0.000**	**-11.9%**	**-10.4%**	0.028	0.058	0.002	Not subst.
**Dimension 4: lack of normative integration**									
14 I give to good causes	**0.000**	0.007	**0.000**	2.2%	2.6%	0.006	0.087	0.001	Not subst.
15 I sometimes do something for my neighbours	0.183	0.001	0.112	-0.3%	-0.4%	0.001	0.313	0.000	None
16 I put glass items in the glass recycling bin	**0.000**	0.023	**0.000**	-1.2%	-2.2%	0.020	**0.000**	0.003	Not subst.
17 Work is just a way of earning money	**0.000**	0.014	**0.000**	-1.5%	-4.1%	0.010	**0.000**	0.004	Not subst.

* Model 1: $Y = \beta_0 + \beta_1 M$; Model 2: $Y = \beta_0 + \beta_1 M + \beta_2 G$; Model 3 : $Y = \beta_0 + \beta_1 M + \beta_2 G + \beta_3 M*G$. Y=item, M=matching variabele=dimensions scale en G=grouping variable=Turkish vs native Dutch.

** Results in bold if the following criteria for DIF were met: P value < 0.001, % difference in β > 10% [39], Nagelkerke pseudo R^2 Δ >= 0.035 [40].

*** None: P value total DIF >= 0.001; Not substantial: P value total DIF < 0.001 & Δ R^2 (M3-M1) < 0.035.
