# Supplementary material for: Evaluating the cross-cultural validity of the Dutch version of the Social Exclusion Index for Health Surveys (SEI-HS): A mixed methods study
Source: PLoS One. 2019 Nov 5;14(11):e0224687. doi: 10.1371/journal.pone.0224687 (PMC6830809; doi:10.1371/journal.pone.0224687)
Supplement: S2 Table — (PDF) [file pone.0224687.s002.pdf]

S2 Table. Factor loadings items SEI-HS in adults of Surinamese, Moroccan and Turkish origin compared to the reference values in the general Dutch population #.

	Surinamese		Moroccan		Turkish		Reference
**Dimension 1: Limited social participation**							
Item 1	*0.773*	*.000*	*0.734*	*.000*	*0.825*	*.000*	0.769
Item 2	*0.497*	*.000*	*0.481*	*.000*	*0.551*	*.000*	0.504
Item 3	*0.561*	*.000*	*0.570*	*.000*	*0.558*	*.000*	0.479
Item 4	*0.780*	*.000*	*0.727*	*.000*	*0.789*	*.000*	0.769
Item 5	*0.770*	*.000*	*0.752*	*.000*	*0.718*	*.000*	0.689
Item 6	*0.151*	*.000*	*0.304*	*.000*	*0.217*	*.000*	0.258
**Dimension 2: material deprivation**							
Item 7	*0.650*	*.000*	*0.596*	*.000*	*0.628*	*.000*	0.588
Item 8	*0.546*	*.000*	*0.591*	*.000*	*0.594*	*.000*	0.519
Item 9	*0.723*	*.000*	*0.567*	*.000*	*0.603*	*.000*	0.720
Item 10	*0.767*	*.000*	*0.722*	*.000*	*0.680*	*.000*	0.679
**Dimension 3: inadequate access to basic social rights**							
Item 11	*0.380*	*.000*	*0.384*	*.000*	*0.312*	*.000*	0.435
Item 12	*0.393*	*.000*	*0.440*	*.000*	*0.495*	*.000*	0.436
Item 13	*0.252*	*.000*	*0.262*	*.000*	*0.149*	*.000*	0.233
**Dimension 4: lack of normative integration**							
Item 14	*0.440*	*.000*	*0.453*	*.000*	*0.487*	*.000*	0.414
Item 15	*0.379*	*.000*	*0.528*	*.000*	*0.578*	*.000*	0.332
Item 16	*0.229*	*.000*	*0.257*	*.000*	*0.241*	*.000*	0.336
Item 17	*0.191*	*.000*	0.097	.052	-0.046	.299	0.298

Confirmatory Factor Analysis in SPSS AMOS
