# Supplementary material for: Evaluating the cross-cultural validity of the Dutch version of the Social Exclusion Index for Health Surveys (SEI-HS): A mixed methods study
Source: PLoS One. 2019 Nov 5;14(11):e0224687. doi: 10.1371/journal.pone.0224687 (PMC6830809; doi:10.1371/journal.pone.0224687)
Supplement: S1 Appendix — (PDF) [file pone.0224687.s003.pdf]

S1 Appendix. Dutch version of the SEI-HS.

Dimensie 1: Onvoldoende sociale participatie

Er volgen nu enkele uitspraken. Wilt u van elk van de volgende uitspraken aangeven in hoeverre die op u, zoals u **de laatste tijd** bent, van toepassing is?

	*Kruis op iedere regel uw antwoord aan **	ja	min of meer	nee
a.	Er is altijd wel iemand in mijn omgeving bij wie ik met mijn dagelijkse probleempjes terecht kan.	❑	❑	❑
b.	Ik ervaar een leegte om mij heen.	❑	❑	❑
c.	Er zijn genoeg mensen op wie ik in geval van narigheid kan terugvallen.	❑	❑	❑
d.	Ik mis gezelligheid om mij heen.	❑	❑	❑
e.	Vaak voel ik me in de steek gelaten.	❑	❑	❑

Hoe vaak hebt u contact met buren of mensen die bij u in de straat wonen?

- ☐ minstens 1 keer in de week
- ☐ 3 keer per maand
- ☐ 2 keer per maand
- ☐ 1 keer per maand
- ☐ minder dan 1 keer per maand
- ☐ zelden of nooit

Dimensie 2: Materiële deprivatie

Heeft uw huishouden meestal voldoende geld om de volgende dingen te doen?

	ja	nee
a. uw huis goed verwarmen	☐	☐
b. lidmaatschap van sportclub of vereniging betalen	☐	☐
c. bij vrienden of familie op visite gaan	☐	☐

Heeft u de **afgelopen 12 maanden** moeite gehad om van het inkomen van uw huishouden rond te komen?

- ☐ Nee, geen enkele moeite
- ☐ Nee, geen moeite, maar ik moet wel opletten op mijn uitgaven
- ☐ Ja, enige moeite
- ☐ Ja, grote moeite

Dimensie 3: Onvoldoende toegang tot sociale grondrechten & Dimensie 4: Onvoldoende normatieve integratie

Hieronder wordt een aantal stellingen gegeven. Wilt u aangeven in hoeverre u het eens bent met deze stellingen?

Kruis op iedere regel uw antwoord aan.	helemaal eens	beetje eens	niet eens/ niet oneens	beetje oneens	helemaal oneens
a. De mensen in mijn buurt kunnen in het algemeen slecht met elkaar opschieten.	☐	☐	☐	☐	☐
b. Werken is slechts een manier om geld verdienen.	☐	☐	☐	☐	☐

Heeft u of iemand in uw huishouden de **afgelopen 12 maanden** een medische behandeling of tandheelkundige behandeling nodig gehad, maar deze niet ontvangen?

☐ ja
☐ nee

Wat geldt voor u?

Ik geef geld aan goede doelen

☐ ja
☐ nee

Ik doe af en toe iets voor de buren

☐ ja
☐ nee

Ik breng glas naar de glasbak

☐ ja, altijd
☐ ja, soms
☐ nee, nooit

Hoe tevreden bent u met uw woning?

Druk dit uit in een rapportcijfer van 1 tot en met 10, 1=zeer ontevreden, 10=zeer tevreden

1	2	3	4	5	6	7	8	9	10
☐	☐	☐	☐	☐	☐	☐	☐	☐	☐

>>> De vragen mogen verspreid in de vragenlijst geplaatst worden, bij voorkeur in samenhang met vergelijkbare onderwerpen.
